# FGF-2, TGFβ-1, PDGF-A and respective receptors expression in pleomorphic adenoma myoepithelial cells: an *in vivo* and *in vitro* study

**DOI:** 10.1590/S1678-77572010000100014

**Published:** 2010

**Authors:** Lucyene MIGUITA, Elizabeth Ferreira MARTINEZ, Ney Soares de ARAÚJO, Vera Cavalcanti de ARAÚJO

**Affiliations:** 1 DDS, MSc, Department of Oral Pathology, São Leopoldo Mandic Institute and Research Center, Campinas, SP, Brazil.; 2 DDS, MSc, PhD, Department of Oral Pathology, São Leopoldo Mandic Institute and Research Center, Campinas, SP, Brazil.

**Keywords:** Myoepithelial cells, Pleomorphic adenoma, Growth factors, FGF-2, TGFβ-1, PDGF-A

## Abstract

Myoepithelial cells have an important role in salivary gland tumor development, contributing to a low grade of aggressiveness of these tumors. Normal myoepithelial cells are known by their suppressor function presenting increased expression of extracellular matrix genes and protease inhibitors. The importance of stromal cells and growth factors during tumor initiation and progression has been highlighted by recent literature. Many tumors result from the alteration of paracrine growth factors pathways. Growth factors mediate a wide variety of biological processes such as development, tissue repair and tumorigenesis, and also contribute to cellular proliferation and transformation in neoplastic cells.

**Objectives:**

This study evaluated the expression of fibroblast growth factor-2 (FGF-2), transforming growth factor β-1 (TGFβ-1), platelet-derived growth factor-A (PDGF-A) and their respective receptors (FGFR-1, FGFR-2, TGFβR-II and PDGFR-α) in myoepithelial cells from pleomorphic adenomas (PA) by in vivo and in vitro experiments.

**Material and Methods:**

Serial sections were obtained from paraffin-embedded PA samples obtained from the school’s files. Myoepithelial cells were obtained from explants of PA tumors provided by surgery from different donors. Immunohistochemistry, cell culture and immunofluorescence assays were used to evaluate growth factor expression.

**Results:**

The present findings demonstrated that myoepithelial cells from PA were mainly positive to FGF-2 and FGFR-1 by immunohistochemistry and immunofluorescence. PDGF-A and PDGFR-α had moderate expression by immunohistochemistry and presented punctated deposits throughout cytoplasm of myoepithelial cells. FGFR-2, TGFβ-1 and TGFβR-II were negative in all samples.

**Conclusions:**

These data suggested that FGF-2 compared to the other studied growth factors has an important role in PA benign myoepithelial cells, probably contributing to proliferation of these cells through the FGFR-1.

## INTRODUCTION

Myoepithelial cells are important components of benign and malignant salivary gland tumors contributing to histological diversity and low grade pattern of these tumors^2,5,6^. It is known that normal myoepithelial cells have an important role as tumor suppressors, being therefore a defense against cancer progression^5,42^.

Pleomorphic adenoma (PA) is the most common type of benign salivary gland tumor in both major and minor salivary glands being a good source of myoepithelial cells, different from breast gland tumors^4^.

Several growth factors are involved in the initiation and progression of tumors, as autocrine and paracrine mediators. These include the family of fibroblast growth factor (FGF), transforming growth factor β (TGFβ) and platelet-derived growth factor (PDGF), which are predominant stimulators of cell proliferation and present in the pathogenesis of many tumors, including salivary gland tumors^12,21,26,27,32,43,48^.

The FGF2 also referred as basic FGF (FGFb), is a member of 22 polypeptides localized in the extracellular matrix (ECM), cytoplasm and nucleus of the cells^11,21^. Several functions are attributed to this growth factor such as: mitogenic function, cell differentiation, angiogenesis, phenotypic transformation^3,47^, and survival of tumor and stem cells^13,14,33,46^. In normal myoepithelial cell and myoepithelial-like cell lines of mammary gland, FGF2 is considered to be a product derived from these cells^19,37^, and its enhanced expression is associated to the differentiation of epithelial cells into myoepithelial-like phenotype^19^. The FGF transmembrane receptors FGFR-1 or Flg and FGFR-2 or Bek are required in the development of many tissues, including salivary gland^15,17,28,31^.

The PDGF is a family of five cationic homoand heterodimer isoforms, considered a product of platelet cells synthesized by different cell types^1^. Its synthesis is in response to external stimuli, such as exposure to low oxygen tension^1,12^ or stimulation by other cytokines and growth factors^1^. It has an important role as an autocrine growth factor for PDGF receptorpositive tumor cells^16,38^, but it is poorly elucidated in salivary gland tumors. This factor exerts its biologic effects by inducing homo- or heterodimeric complexes of α- and β- tyrosine kinase receptors, PDGFR-α and PDGFR-β^1,16^. Both receptors can activate signal transduction pathways, stimulating cell growth and angiogenesis, whereas activation of the PDGFRα inhibits and stimulates chemotaxis of certain cell types^1^.

TGFβ is a highly pleiotropic cytokine present in mammals that modulates proliferation, differentiation, apoptosis, adhesion, and migration of various cell types and favors the production of ECM proteins^36^. Production of TGFβ is part of the regulatory mechanism controlling the growth and differentiation of both nonmalignant and malignant cells^34^. TGFβ-1 initiates intracellular signaling by two types of transmembrane receptors known as type I (TGFβRI) and type II (TGFβRII) receptors^7,34^.

Based on the role of growth factors in tumors, the aim of this study was to analyze the expression of FGF-2, TGFβ-1, PDGF-A, and their respective receptors (FGFR-1, FGFR-2, TGFβR-II and PDGFR-α) on benign myoepithelial cells from PA *in vivo* by immunohistochemistry and also *in vitro* by immunofluorescence.

## MATERIAL AND METHODS

### Immunohistochemistry

The research protocol was approved by the Research Ethics Committee of São Leopoldo Mandic Institute and Research Center, Campinas, Brazil (Protocol # 07/124).

Twelve cases of PA were retrieved from the files of the Department of Pathology, São Leopoldo Mandic Institute and Research Center, Campinas, Brazil (Figure 1).

**Figure 1:** Sex, age and localization of the pleomorphic adenoma

**Case**	**Gender**	**Age (years)**	**Localization**
1	Male	20	Upper Lip
2	Female	*	Upper Lip
3	Female	30	Upper Lip
4	Female	22	Submandibular region
5	Female	23	Parotid
6	Female	28	Hard Palate
7	Female	56	Upper Lip
8	Female	25	Upper Lip
9	Female	36	Hard Palate
10	Female	25	Upper Lip
11	Female	28	Hard Palate
12	Female	39	Palate

* Not available.

Three-micrometer-thick serial sections were obtained from paraffin-embedded samples and the dewaxed sections were processed to antigen retrieval. Endogenous peroxidase was blocked by incubation with 3% hydrogen peroxide and methanol (1:1). After washing, sections were incubated with primary polyclonal antibodies (Figure 2). Signal detection was performed using the DAKO EnVision Peroxidase (DakoCytomation, Carpentaria, CA, USA), followed by a diaminobenzidine chromogen solution and counterstaining with Mayer’s hematoxylin. The reactions were executed by Dako Autostainer Plus (DakoCytomation).

**Figure 2:** Primary polyclonal antibodies

**Antibody**	**Immunohistochemical Dilution**	**Immunofluorescence Dilution**	**Host**	**Sources**
FGF-2	1:100	1:50	Rabbit	St. Cruz Biotechnology^1^
FGFR-1	1:150	1:100	Rabbit	St. Cruz Biotechnology^1^
FGFR-2	1:50	1:50	Rabbit	St. Cruz Biotechnology^1^
TGFβ-1	1:200	1:100	Rabbit	St. Cruz Biotechnology^1^
TGFβR-II	1:50	1:50	Rabbit	St. Cruz Biotechnology^1^
PDGF-A	1:50	1:50	Rabbit	St. Cruz Biotechnology^1^
PDGFR-α	1:100	1:50	Rabbit	St. Cruz Biotechnology^1^
Vimentin	1:300	1:300	Mouse	Dako^2^
α-smooth muscle actin	1:300	1:50	Mouse	Dako^2^
Calponin	1:50	1:20	Mouse	Dako^2^
CK7	1:100	1:50	Mouse	Dako^2^

1 Santa Cruz Biotechnology, Inc., Santa Cruz, CA, USA.

2 DakoCytomation, Carpentaria, CA, USA.

The labeled sections were qualitatively evaluated by two examiners observing cytoplasm and/or nuclear positive stained cells. The immunohistochemical reaction was evaluated according to the extent of positive staining using the following score, by percentage: 0, staining from 0 to 10%; 1, staining from 10 to 25%; 2, staining from 25 to 50%; 3, staining up to 50%.

### Cell Culture

Myoepithelial cells were obtained from explants of PA tumors (cases 4, 5 and 8) provided by surgery from different donors. This part of the study was conducted after approval of the Research Ethics Committee of São Leopoldo Mandic Institute and Dental Research Center, Campinas, Brazil (Protocol # 2009/0014).

**Figure 4:**
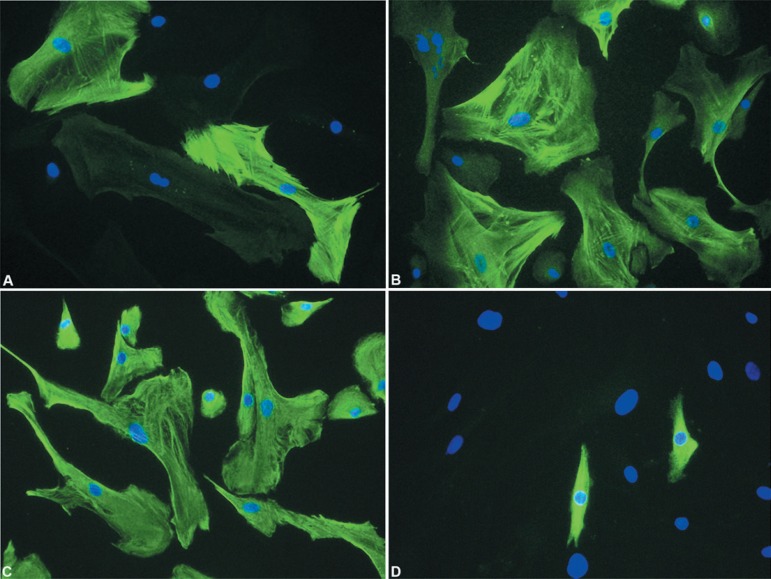
Immunostaining for α-AML (A), calponin (B), vimentin (C) and CK7 (D) in myoepithelial cells from PA. Observe that some myoepithelial cells were negative for α-AML (A) and calponin (B), but all cells were immunoreactive for vimentin (C). Rare cells expressed CK-7 (D). Nuclei stained with DAPI appear in blue. Original magnification- A-D: ×200

The obtained cells were cultured in Dulbecco’s modified Eagle medium (DMEM, Sigma-Aldrich Inc., St Louis, MO, USA) supplemented by 1% antimycotic-antibiotic solution (10000 units penicillin, 10 mg streptomycin and 25 µg amphotericin B per mL in 0.9% sodium chloride; Sigma^®^), containing 10% of fetal bovine serum (FBS; Gibco, Buffalo, NY, USA), plated in 60-mm diameter plastic culture dishes and incubated under standard cell culture conditions (37°C, 100% humidity, 95% air, and 5% CO_2_). When the cells reached confluence, they were detached with 0.05% trypsin and subcultured at a density of 20,000 cells/well (~110 cells/mm^2^). The cells were used at subculture levels 3 or 4, and the cells were characterized using anti-α smooth muscle actin, anti-calponin and anti-vimentin (Figure 4 A-C). CK7 was also analyzed (Figure 4 D). The primary polyclonal antibodies are described at Figure 2.

**Figure 3:** Immunohistochemical expression of FGF-2, PDGF-A, TGFβ-1 and respective receptors in myoepithelial cells of pleomorphic adenoma

**Case**	**FGF-2**	**FGFR-1**	**FGFR-2**	**TGFβ-1**	**TGFβR-2**	**PDGF-A**	**PDGFR-α**
1	3	2	0	0	0	0	0
2	3	3	0	0	0	0	1
3	3	3	0	0	0	1	1
4	3	2	1	0	0	1	1
5	3	3	1	0	0	1	1
6	3	2	0	0	0	0	1
7	3	2	0	0	0	0	1
8	3	3	0	0	0	1	1
9	3	3	2	0	0	1	1
10	3	3	1	0	0	1	1
11	3	3	1	0	0	0	0
12	3	3	1	0	0	0	0

Score 0: 0- 10% of positive cells; Score 1: 10- 25% of positive cells; Score 2: 25- 50% of positive cells; Score 3: up to 50% of positive cells.

### Immunofluorescence

Cells grown on coverslips were fixed in methanol for 6 min at 20°C, rinsed in PBS followed by blocking with 1% bovine albumin in phosphate buffer saline (PBS) for 30 min at room temperature. The primary polyclonal antibodies are described at Figure 2. Control staining reaction was performed using PBS as nonimmune IgGs at the same dilution used for the primary antibody. The secondary antibodies used were biotinylated anti-rabbit and anti-mause IgG (Vector Laboratories Inc, Burlingame, CA, USA). Fluorescein-streptavidin conjugated (Vector) were used for the second step. After washing, preparations were mounted using Vectashield DAPI-associated (4'-6-diamidino-2-phenylindole) (Vector) and observed on a Zeiss Axioskop 2 conventional fluorescence microscope (Zeiss, Carl Zeiss MicroImaging, Oberköchen, Germany) equipped with ×63 Plan Apochromatic 1.4NA and ×100 Plan Apochromatic 1.4NA objectives in standard conditions (Zeiss^®^).

## RESULTS

### Immunohistochemistry

**Figure 5:**
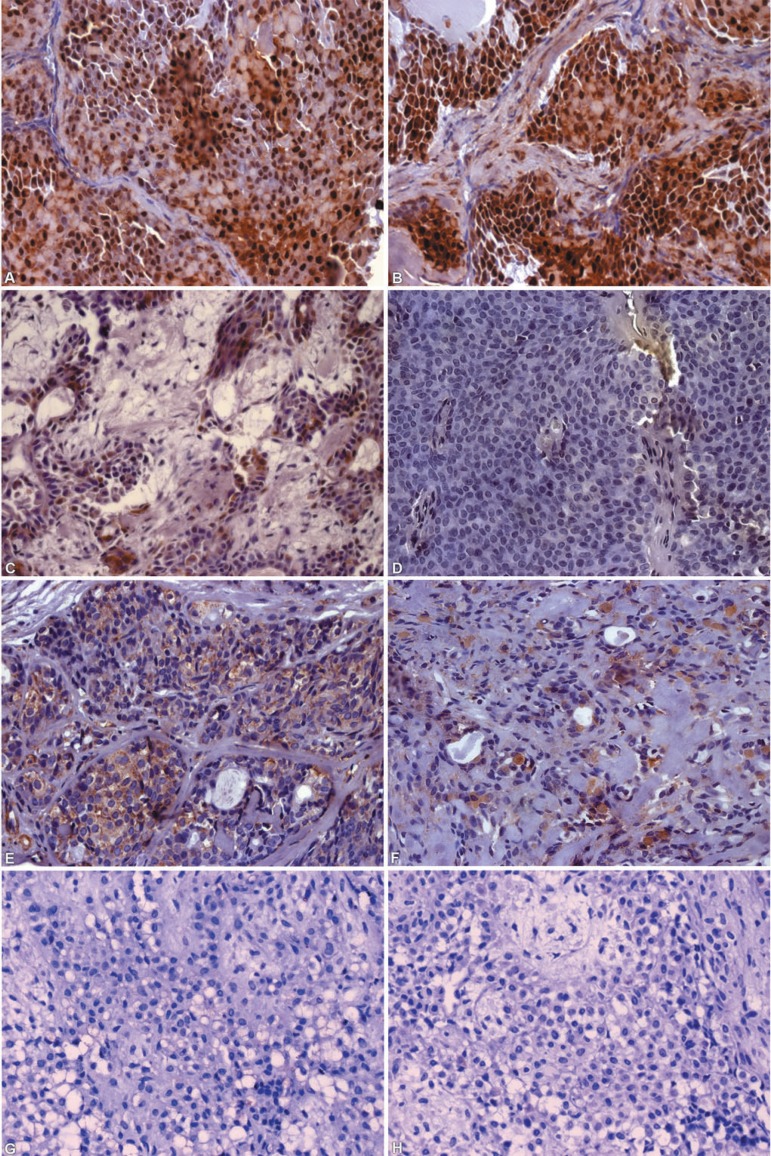
Immunohistochemical expression of FGF-2 (A and B), FGFR-1 (C), FGFR-2 (D), PDGF-A (E), PDGF-α (F), TGF- β (G) and TGFβR-II (H). Observe that most myoepithelial cells were strongly positive for FGF-2 (A and B), while for FGFR- 1 only some cells were immunostained (C). No expression was observed for FGFR-2 (D). PDGF-A (E) and PDGFR-α (F) were moderately immunoreactive in some cytoplasm and nuclei of myoepithelial cells. No reaction for TGF-β (G) and TGFβR-II (H) was observed. Original magnification- A-H: ×400

FGF-2 was strongly expressed in most cytoplasms and nuclei of PA myoepithelial cells (Figure 5A and B). FGFR-1 was immunoreactive in some cytoplasm and nucleus (Figure 5C). On the other hand, there was no FGFR-2 expression (Figure 5D) except for focal cells in two cases (data not shown). PDGF-A immunostaining was moderate in the cytoplasm and in some nuclei of myoepithelial cells (Figure 5E) with the same pattern of immunoreaction for PDGFR-α (Figure 5F). TGFβ-1 (Figure 5G) and TGFβR-2 were negative in all studied cases (Figure 5H).

Figure 3 summarizes the expression of the growth factors and their receptors.

### Immunofluorescence

**Figure 6:**
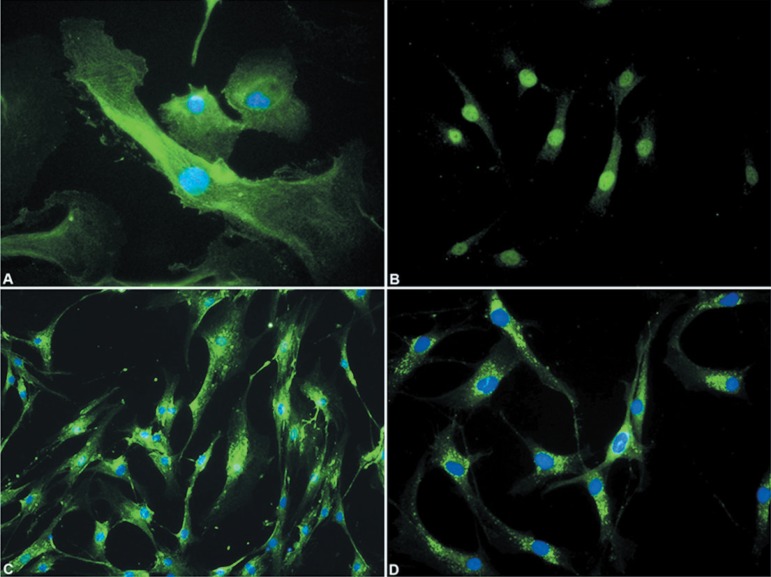
Immunostaining for FGF-2 (A), FGFR-1 (B), PDGF-A (C) and PDGFR-α (D) in myoepithelial cells from PA. FGF- 2 was expressed as a reticular network in all cytoplasm (A). FGFR-1 was immunoreactive mainly in the nuclei of the cells (B). PDGF-A (C) and PDGFR-α(D) were immunoexpressed as punctate deposits throughout the cytoplasm. Nuclei stained with DAPI appear in blue. Original magnification- A-D: ×400

FGF-2 was immunoexpressed in all myoepithelial cells and was detected as a diffuse reticular network throughout the cytoplasm (Figure 6A). FGFR-1 immunostaining all myoepithelial cells, mainly in the nucleus (Figure 6B). PDGF-A (Figure 6C) and PDGFR-α (Figure 6D) were immunoexpressed as punctate deposits throughout the cytoplasm. No immunoreactivity for FGFR-2, TGFβ-1 and TGFβR-II was observed in the myoepithelial cell cultures (data not shown).

## DISCUSSION

The present findings demonstrated that FGF2 and FGFR-1 were the main expressed factors in myoepithelial cells from PA by *in vivo* and *in vitro* experiments compared with the FGFR-2, PDGF-A, PDGFR-α, TGFβ-1 and TGFβR-II.

The benign myoepithelial cell has an important role in salivary gland tumor development. Tumors composed of these cells have low aggressiveness^2^. It is known that normal myoepithelial cells have a suppressor function, presenting increased expression of ECM genes and protease inhibitors and reduced expression of angiogenic factors and proteinases^5,42^.

Pleomorphic adenoma is reported to be a great source of myoepithelial cells^4^. In the present study, this evidence was confirmed by the in vitro characterization of myoepithelial cell line from PA, which presented mainly positive myoepithelial markers (anti-α smooth muscle actin, anticalponin and anti-vimentin) and negative or rare positive cells for luminal markers (CK-7 and AE1/ AE3). In addition, in the present study growth factors that promote the outgrowth of epithelial cells have not been added to the cultures.

In the present study, FGF-2 was strongly expressed in most cytoplasm and nucleus of PA myoepithelial cells by immunohistochemistry. It is known that FGF-2 is an important growth factor involved in cell proliferation^9^ and differentiation^10^. It can be found in ECM, cytoplasm and nucleus of the cells^11,29^ activating signal pathways by transmembrane receptors, acting as an autocrine and paracrine factor^5,26,27^.

The immunofluorescence assay confirmed the reactivity of myoepithelial cells to FGF-2, mainly in the cytoplasm exhibiting a diffuse reticular network. Taverna, et al.^45^ (2008) demonstrated that intracellular trafficking of endogenous FGF2, destined for secretion into the ECM, is related with the presence of actin filament. This might explain the reticular and diffuse expression pattern of this growth factor throughout the cytoplasm. Myoepithelial cells from PA were positive to FGFR-1, by immunohistochemistry assay, in both cytoplasm and nucleus. Nuclear immunoexpression was mainly evident in the *in vitro* assay.

In general, the majority of growth factor receptors play their role in signal transduction at the cell surface, which activates ligand-dependent intracellular signaling networks^35^. However, some studies have demonstrated a different pathway involving nuclear translocation after internalization^8,18,49^.

It is demonstrated that FGFR-1, which is is also a transmembrane protein, translocate to the nucleus after ligand stimulation that is mediate by importin-α and E-cadherin^8,35,41^, playing a role in the regulation of cell cycle. In malignant salivary gland tumors, the overexpression of FGF2 and FGFR-1 facilitates neoplastic progression^21,27^. FGFR-2 expression was negative in all myoepithelial cells both in *in vivo* and *in vitro* results. In the literature, FGFR-2 has been considered as risk factor in breast cancer^24^ and contributes to cell growth, invasiveness, motility and angiogenesis^22,25^. The absence of FGFR-2 in PA is in accordance with the benign behavior of this tumor.

In the present study, no immunoreactivity for TGFβ-1 and TGFβR-II was observed in PA and neither in the myoepithelial cell cultures, which is in accordance with the results of Kusafuka, et al.^20^ (2001).

Numerous studies have demonstrated that TGFβ-1 may strongly inhibit growth and induce apoptosis in nontransformed cells. In malignant tumors, the loss of TGFβ-1 is associated with tumor immunosurveillance^39^. In established tumors, TGFβ-1 exerts a favorable effect for the survival, progression and metastasis mainly related with malignant tumors^30,40^.

PDGF-A immunohistochemical expression was moderate in the cytoplasm and nucleus of some myoepithelial cells with the same pattern of immunoreaction for PDGFR-α. This factor has a paracrine function in PDGFR positive cells and stimulates the stroma to up-regulate FGF-2, promoting angiogenesis and cell proliferation in neoplastic cells^32^.

PDGF is related to malignant transformation, as previously demonstrated. Demasi, et al.^12^ (2008) observed that PDGF-A and PDGFR-α were slightly detected in remnant pleomorphic adenoma presented in CXPA, but they were collectively highly expressed as soon as the malignant phenotype was achieved and they were kept on elevated levels during the progression to the advanced stages of CXPA.

We have also observed that PDGF-A and its receptor, by immunofluorescence, were present as punctate deposits throughout the cytoplasm. The punctate pattern of PDGF-A and PDGFR-αα expression is justified because they regulate intracellular signal transduction by internalization to cytoplasm cell via caveolae endocytosis^23^. Caveolae is flask-shaped plasma membrane invaginations that mediate endocytosis and transcytosis of plasma macromolecules, and also growth factors as PDGF, present in cytoplasm of cells as a punctate pattern^23,44^.

The results obtained both *in vivo* and *in vitro* assays were very similar, demonstrating that FGF2, compared to the other studied growth factors, is an important factor in myoepithelial cells of PA, probably contributing to PA proliferation through the FGFR-1.

## CONCLUSION

FGF-2 may have an important role in PA myoepithelial cell proliferation mediated by FGFR1 receptor.
